# Infant Oral Health Knowledge and Awareness: Disparity among Pregnant Women and Mothers visiting a Government Health Care Organization

**DOI:** 10.5005/jp-journals-10005-1160

**Published:** 2012-12-05

**Authors:** Anup Nagaraj, Sonia Pareek

**Affiliations:** Professor and Head, Department of Public Health Dentistry, Jaipur Dental College, Jaipur, Rajasthan, India; Postgraduate Student, Department of Public Health Dentistry, Jaipur Dental College, Jaipur, Rajasthan, India, e-mail: dr.soniapareek@gmail.com

**Keywords:** Infant feeding, Knowledge, Mothers, Oral health

## Abstract

**Objectives:** The present study is designed to assess the knowledge, attitude and practices of pregnant women and mothers about feeding habits and infant oral health.

**Materials and methods:** A total of 230 study subjects were divided into two groups: Group A included pregnant women and group B were mothers of child up to 1 year of age. Each group comprised of 170 subjects. A self-administered questionnaire comprising of total 23 questions on infant feeding practices, nocturnal bottle feeding, correct age of eruption of first teeth and first dental visit. Two separate questionnaires were framed for both the groups.

**Results:** There was a lack of knowledge among both the groups about infant feeding and weaning. Nocturnal bottle feeding was more prevalent.

**Conclusion:** The present study reflects a need for maternal counseling on infant oral health.

**How to cite this article:** Nagaraj A, Pareek S. Infant Oral Health Knowledge and Awareness: Disparity among Pregnant Women and Mothers visiting a Government Health Care Organization. Int J Clin Pediatr Dent 2012;5(3):167-172.

## INTRODUCTION

Feeding practices are major determinants of both growth and morbidity in infants and young children. Malnutrition in children is more an interplay of female illiteracy, ignorance about nutritional needs of infants and young children and poor access to health care. Appropriate feeding is crucial for the healthy growth and development of an infant. The health and nutritional status of mothers and children are intimately linked.^1^ Breastfeeding promotion is a significant child survival strategy. Interventions to improve early and correct infant feeding practices can result in considerable reduction in neonatal morbidity and mortality.^2^

A new scheme for the protection of women from the risk of maternal mortality, ‘Janani Suraksha Yojana’ was launched by Government of India in April 2005. The first expected outcome covering the period 2005 to 2012 is to reduce infant mortality rate to 30% of 1000 live births. According to latest data it is 64 per 1000 live births in rural and 40 in urban. Infant mortality rate (IMR) in Rajasthan is 74 per 1000 live births. The reason ferreted out behind this huge number is complete lack of awareness within the community about appropriate feeding practices. IMR can be reduced by promoting optimal infant and young child feeding practices. The National Nutrition Policy adopted by the Government of India under the aegis of the Department of Women and Child Development laid due emphasis on nutrition and health education of mothers on infant and young child feeding and efforts to trigger appropriate behavioral changes among the mothers, were considered as direct interventions for reducing malnutrition in children.^3^

While focusing on the health and nutritional status of the infants and young children, it must be noted that the health and nutritional status of mothers and children are intimately linked. Improved infant and young child feeding begins with ensuring the health and nutritional status of women, in their own right, through all stages of life and continues with women as providers for their children and families.^4^

Infant oral health is the foundation upon which preventive education and dental care must be built to enhance the opportunity for lifetime, free of preventable oral diseases. Mothers are decision makers and play an important role in achieving the best oral health outcomes for their young children. A young child’s dental environment is complex because their mothers’ and/or caregiver’s dental knowledge, attitudes, beliefs and practices affect the child’s oral condition.^4,5^ Very young children are dependent on their mothers to attend to their oral hygiene and feed them. Inappropriate bottle use patterns, such as the addition of sweeteners to the liquid and prolonging exposure of sugary liquids at bedtime, and later age at weaning have been linked to early childhood caries.^6^

The state of Rajasthan lacks in adequate facilities for mother and child. The children are suffering from malnutrition and there is an increase in maternal and IMRs. Also the female literacy rate is about 46% and the mothers are more inclined toward the traditional cultural practices of child rearing. Considering mother’s important role in well-being of young children, it is essential to explore their knowledge, attitude and beliefs as it affects the dental care children receive at home and their access to professional dental services. The present study is designed with the aim to assess the infant feeding practices of pregnant females and mothers visiting the Outpatient Department of the Government Hospital in Jaipur city.

## MATERIALS AND METHODS

This cross-sectional, questionnaire study was conducted in ‘Mahila Chikitsalya’, a Government Hospital in Jaipur, after obtaining a prior permission from the concerned authorities. The study sample comprises of the pregnant women and mothers of child up to 1 year of age. The study participants were divided into two groups: Group A included pregnant women and group B were mothers of child up to 1 year of age. Each group comprised of 170 subjects. Subjects who were not willing to participate in the study and mothers of children with congenital anomalies and twins were excluded.

The data was collected by means of a structured interview in a form of questionnaire which was translated into local language and filled by the investigator to prevent bias. The pregnant females were interviewed at the antenatal department and the mothers were interviewed when they use to come for immunization or pediatric visit at the hospital.

Questionnaire was designed to obtain general information, including personal data and the socio- demographic profile. Questions about the history of miscarriage, preterm birth were also recorded. Questions about oral hygiene practices, importance of oral health, causes of tooth decay, correct age of weaning, importance of deciduous dentition, first dental visit were included in first set and common for both the groups. Exclusive questions for group B included dietary information: Breast- feeding/bottle feeding, duration and frequency of feeding, infant formula, sugar content in infant’s diet and use of pacifiers.

A pilot study was carried out to ensure the validity and reliability of the questionnaire. This procedure was done to ascertain the appropriateness of each question, as well as eliciting any feedback from the responders. Ethical clearance was obtained from the Ethical Committee of Jaipur Dental College.

## RESULTS

Epidemiological data was obtained from samples comprising of subjects of different socioeconomic status. The data was analyzed using descriptive statistics and Chi-square test was carried out to assess association across groups. The majority of mothers in this study were from low-income families, mostly young adults, majority housewives; almost 50% were primigravidae (Table 1).

**Table 1:** **Table 1:** Distribution of study participants-based on demographic data and general information

		*Group A (%)*		*Group B (%)*	
*Age*					
16-20 years		22.9		10	
21-25 years		51.1		30	
26-30 years		20.5		40	
Above 30 years		5.2		20	
*Distribution of subjects according to number of children*					
Primigravidae		57.6		61.1	
One child		31.1		28.8	
More than 2		1.7		10	
*Level of education*					
Illiterate		21.1		20.5	
Middle and secondary education		40		30	
Senior secondary, graduate and above		38.8		49.4	
*Occupation*					
Housewife		79.4		75.8	
Employed		20.5		24.1	
*Annual income*					
Less than 1 lac per annum		49.4		33.5	
1-2 lac per annum		18.8		16.4	
2-4 lac per annum		18.2		37	
More than 4 lac per annum		13.5		12.9	
*Family size*					
Joint family		54.7		32.3	
Nuclear family		45.2		67.6	
*History of miscarriage*		10.5		9.4	

### Knowledge on Causes of Tooth Decay

Nearly half of the subjects (48.8%) from both the groups believed that increased sugar consumption leads to dental caries. A total of 32.9 and 40% participants from groups A and B respectively considered that incorrect tooth brushing leads to dental caries.

### Oral Cleansing Methods

**Fig. 1:**
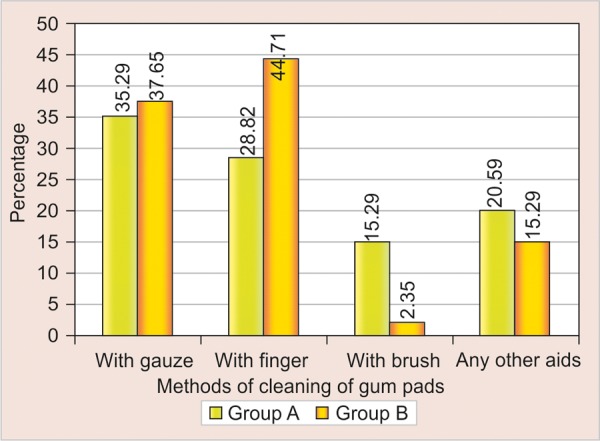
Methods of cleaning of gum pads

Overall, 35.2 and 37.6% subjects from both the groups believed that the gum pads of the children should be cleaned using gauze. Cleaning with finger was practiced by 44.71% of group A and 28.82% of group B subjects. Brushing was supported least by both the groups (Table 2, Fig. 1).

**Table 2:** **Table 2:** Methods of cleaning of gum pads

*Methods of cleaning of gum pads*		*Group A*				*Group B*			
		* No.*		* Percentage*		* No.*		* Percentage*	
With gauze		60		35.29		64		37.65	
With finger		49		28.82		76		44.71	
With brush		26		15.29		4		2.35	
Any other aids		35		20.59		26		15.29	
Total		170		100.00		170		100.00	

### Eruption of Teeth

Respondents of group B had better knowledge (67%) about correct age for eruption of first tooth as compared with group A (49.41%) (Table 3).

### Myths Associated with Natal Teeth

When the study participants were inquired about the myths associated with natal teeth it was found that participants from both the groups considered natal teeth as bad omen. The respondents relate the presence of natal tooth with supernatural powers, ill-luck and were in belief that the child will bring misfortune to the family.

### First Dental Visit

The knowledge about the child’s first dental visit was poor among both the groups, when they were asked about the first dental visit only about 22.3% from group A and 25.8% from group B reported that the child should be taken to the dentist for the first time when the first tooth erupts in the oral cavity. About 34.7% from group A and 29.4% from group B reported that the child should be taken to the dentist only when there is pain (Table 4).

**Table 3:** **Table 3:** Knowledge about age of eruption of first tooth

*Age of eruption of first tooth*		*Group A*				*Group B*			
		* No.*		* Percentage*		* No.*		* Percentage*	
At 6 month		84		49.41		115		67.65	
After 1 year		57		33.53		46		27.06	
Do not know		29		17.06		9		5.29	
Total		170		100.00		170		100.00	

**Table 4:** **Table 4:** Knowledge about child’s first dental visit

*First dental visit*		*Group A*				*Group B*			
		* No.*		* Percentage*		* No.*		* Percentage*	
When the first tooth erupt		38		22.35		44		25.88	
Only when there is a pain		59		34.71		50		29.41	
Do not know		50		29.41		64		37.65	
Not required		23		13.53		12		7.06	
Total		170		100.00		170		100.00	

### Need for Oral Health Counseling

Majority of participants from both the groups (88%) expressed that infant oral health counseling should be provided to the mothers.

### Feeding Practices

It was convincing to know that exclusive breastfeeding was practiced by 83% where as less than 10% subjects fed their child with bottle.

**Fig. 2:**
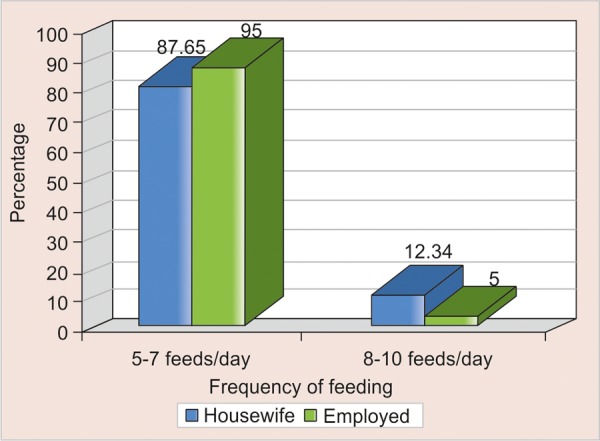
Comparison of feeding frequency of child as provided by working mothers and housewives

**Fig. 3:**
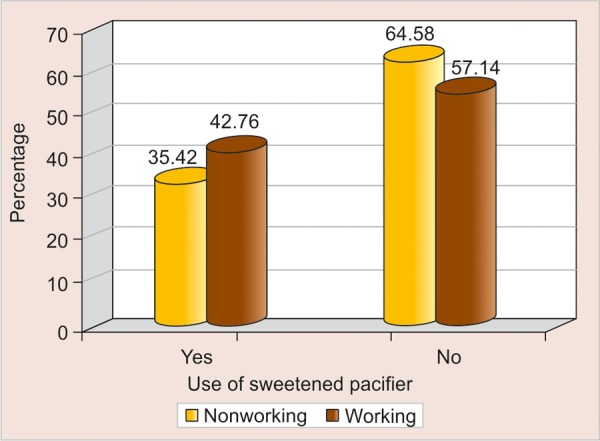
Use of sweetened pacifier by working mothers and nonworking mothers

Optimum feeding (according to infant feeding guidelines) of 8 to 10 times/day was followed by 12.3% of housewives and only 5% of employed subjects (Table 5, Fig. 2). Nearly half of working mothers (42.8%) were interested in using sweetened pacifier whereas just one-third housewives (35.4%) supported this practice (Table 5, Fig. 3). Sixty percent of working mothers fed their children with sugar containing milk/fluids but 55% housewives had a similar way of feeding. About 58.3% of housewives and 52.4% working mothers were practicing nocturnal bottle feeding (Table 5).

**Table 5:** **Table 5:** Comparison of infant feeding practices of working mothers and housewives

		*Working mothers* *(%)*		*Housewives* *(%)*	
Optimum feeding (8-10 times)		5		12.3	
Use of sweetened pacifier		42.8		35.4	
Sugar containing milk/fluid		60		55	
Nocturnal bottle feeding		52.4		58.3	

When the mothers were inquired about the duration for changing the feeding bottle, 75% of housewives and 71% of working mothers were in the habit of changing the feeding bottle in every 3 months (Table 5).

It was surprising to know that inspite of the level of education of mothers about 60% of them were feeding the child with liquids containing sugars. Only 26% of educated mothers use to check the food labels for nutritive value and sugar content in the food supplements they were providing to their child.

## DISCUSSION

It is essential to assess the knowledge and attitudes of the mothers and expectant mothers concerning different issues related to infant feeding and oral health. This will enable us to implement appropriate programs to improve the health of both women and children.

### Oral Health Habits

It was encouraging to find that the majority of mothers/ pregnant females were cleaning their child’s mouth using cotton gauze. The findings of present study were in accordance with another work by SC Chan, JSJ Tsai and NM King^7^ in which cleaning was carried out using a piece of cotton gauze, a handkerchief, a cotton bud or a cotton tipped applicator (Table 2). Respondents from both the group considered that primary teeth were important in the oral cavity.^3^ The findings were contradictory to results by ES Davenport et al^8^ in which primary teeth had no particular function.

Not many of the mothers could recall the time when their child’s first tooth erupted into the mouth. This was inspite of it probably being a momentous event at the time. These findings, to a certain extent, reflect the low awareness of the oral health of the children by their mothers.

The preventive goals during an early dental visit may include improvement of oral hygiene and eating habits, informing parents about the risks posed by nonnutritive sucking for development of malocclusions, educating parents regarding traumatic injuries and how to seek emergency care. The ultimate aim is to educate and motivate the parents to take all measures to promote oral hygiene and early dental diseases.^9^ First dental visit should be made at 1 year of age for all children from a low socioeconomic background.^10^ The knowledge about the child’s first dental visit was poor among both the groups. The above findings are in accordance with the study by SC Chan, JSJ Tsai and NM King.^7^ The results of the present study differed from the findings by Retna Kumari N et al^11^ where majority were aware of the correct age for the first dental visit. The reason for this difference can be the low literacy rate in Rajasthan where the present study was conducted. This pattern of behavior may indicate barriers to dental services and utilization which needs to be explored in future studies (Table 3).

It was satisfying to find that the majority of mothers/ pregnant females were interested in receiving infant oral health counseling. The need for oral health education for parents was confirmed by the results of this study. This may be due to the fact that health care instructions to parents and children often takes place in dental offices, with limited time and in connection with provision of radical treatment, such as tooth extraction.^7^

### Feeding Habits

Breastfeeding provides multiple nutritional, immunological and psychological benefits to the infant in its first year of life. WHO recommends that infants be exclusively breast- fed for the first 6 months of life, with some breastfeeding continuing for up to 2 years of age. When provided along with appropriate and adequate complementary food, breast milk continues to be an important source of nutrition and provides immunological benefits even after 6 months of age.^12^ Breastfeeding is the commonly accepted practice. The prevalence of children who were breastfed was reported in about 83.9% and bottle feeding in 8.9% subjects as there was an influence of cultural and ethnic factors.

**Fig. 4:**
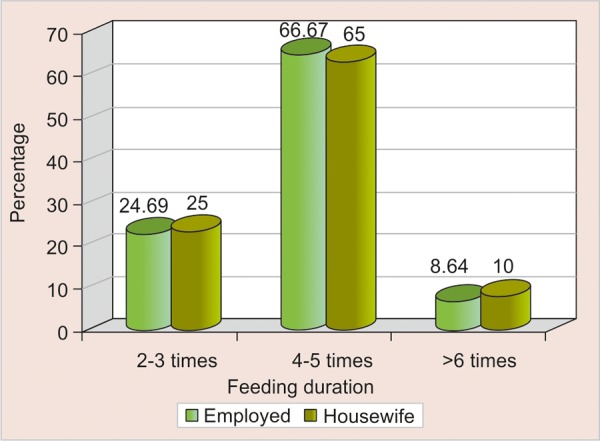
Comparison of feeding duration of child as provided by housewives and working mothers

In the present study when frequency of breastfeeding was compared with occupational status of mothers it was found that housewives were providing the child with the optimum feeds of 8 to 10 times per day (as per the infant feeding guidelines) when compared with working mothers, who were more inclined toward use of sweetened pacifier and bottle feeding (Fig. 4).

Bottle feeding is a major risk factor for development of early childhood caries; the contents of the feeding bottle play a significant role in developing dental caries. Bottle feeding was reported by 8.9% which was contradictory to the findings by Tyagi R^13^ and SC Chan, JSJ Tsai and NM King^7^ in which bottle feeding was practiced in 51 and 33.7% of children respectively.

The alarming finding concerning the existence of the burden of nocturnal bottle feeding (Table 5); there is a need to provide mothers with instructions and guidance in feeding practices. Information on practical ways to control the bedtime feeding practices of young children needs to be made available. The importance of feeding needs to be emphasized prior to the establishment of such a deleterious habit. These nocturnal feeding habits are well known to contribute to caries development in young children. A nursing bottle at night may be used as a form of comforter thus, creating a habit that is subsequently difficult to break.

When the habit of nocturnal bottle feeding was related to the occupation of mother it was found that more housewives as compared with working mothers were in the habit to bottle-feed the child at night. Whenever, the child cries at night the mothers provide them with feeding bottle. The findings are in accordance with the study by Tyagi R,^13^ SCL Chan, JSJ Tsai and NM King,^7^ Ana Paula Pires dos Santos et al^14^ and contradictory with Amjad H Wyne.^15^

The low prevalence of sweetening the pacifier was a positive finding because the use of sweetened pacifier is an established factor in etiology of nursing caries. The association of sweetened pacifier use and its relation with occupation of mother, it was found that 42.8% of working mothers were providing the child with sweetened pacifier. In the absence of mother the child were provided with pacifier (Table 5, Fig. 4). This is similar to the level reported by SCL Chan, JSJ Tsai and NM King^7^ where about 35.6% mothers were providing the child with sweetened dummy. In a study by Amjad H Wyne^15^ about 67.6% were given sweetened pacifier for the age group from birth to 36 months, about 35.1% children were provided sweetened pacifier during night. Both honey as well as sugar was used as sweetening agents for pacifier.

Personal, communal, cultural and economic factors influence dental health behaviors and nutritional habits of the families seeking dental care.^16^ Parents need to be helped to realize that they are role models for their children and to be encouraged to improve the children’s dental health habits.^17^

This report presents a valid contribution to promote breastfeeding. Ideally, it would be best if children were only breastfed and used neither the bottle nor the pacifier. Infant feeding practices, diet and oral hygiene habits plays a crucial role in preventing various dental diseases of the child. Although the limitations of cross-sectional data must be kept in mind, if confirmed by further research, the brief measures presented here hold promise for the rapid and accurate assessment of maternal cognitions that influence oral health promoting behaviors. Such cognitions are potentially modifiable, and can be enhanced through educational and cognitive behavioral interventions.

## SUMMARY

Cultural influences, competing pressures and perceptions of hereditary influences, together with a lack of contemporary oral health knowledge are the main factors affecting oral health knowledge and beliefs. Mother’s knowledge, beliefs and practices helps in formulation of more effective strategies to benefit infants. Mothers were aware about the correct age of eruption of first teeth as compared with pregnant women. Knowledge about first dental visit was lacking among both the groups. Mothers should be educated about the ill effects of nocturnal bottle feeding, in particular with sweetened fluids, and prolonged bottle feeding. Further research is required to identify high- risk parents and target these parents for efficient oral health promotion. The present study, has identified several factors that need consideration in the further exploration and development of primary care physician’s role.

## LIMITATIONS

- This being a questionnaire study has certain limitations of its own
- Comparison was not made among the females who were pregnant for second or third time
- This being a population-based survey with a small sample size, the results cannot reflect on the total mother- child population.

## RECOMMENDATIONS

- Mothers need to be educated about feeding patterns of infants and young children. This guidance may come from health professionals, such as nutritionists, pediatric dentists, general dentists, general medical practitioners and nursing staff of local mother and child health care centers.
- Public health centers should provide oral health counseling for expectant parents. Preventive oral health programs should be carried on for impoverished children. Encourage mothers to initiate and continue breastfeeding.
- There is a necessity to implement anticipatory guidance for oral health in the health care system in Jaipur city.
